# Supplementary material for: The Systems Biology Research Tool: evolvable open-source software
Source: BMC Syst Biol. 2008 Jun 29;2:55. doi: 10.1186/1752-0509-2-55 (PMC2446383; doi:10.1186/1752-0509-2-55)
Supplement: Additional file 1 — SBRT Archive. An archive of the current version of the Systems Biology Research Tool. [file 1752-0509-2-55-S1.zip › sbrt-1.4.0/doc/developers_guide/api/sbrt/Sbrt.html]

Sbrt


|  |  |  |
| --- | --- | --- |
| **Overview** \| **Package** \| **Class** \| **Use** \| **Tree** \| **Deprecated** \| **Index** \| **Help** | |  |
| **PREV CLASS**   NEXT CLASS | **FRAMES**    **NO FRAMES**     **All Classes** |  |
| SUMMARY: NESTED \| FIELD \| CONSTR \| METHOD | DETAIL: FIELD \| CONSTR \| METHOD |  |


---


## sbrt Class Sbrt

```
java.lang.Object
  sbrt.Sbrt
```

---

``` public final class Sbrt extends java.lang.Object ```

This class is used to execute the Systems Biology Research Tool as an
application.

**Author:**
:   This class was written and documented by Jeremiah Wright while in the
    Wagner lab.

---

| **Field Summary** | |
| --- | --- |
| `static java.lang.String` | `GUI_ARG`             The argument used to launch the GUI from the command line. |
| `static int` | `MAJOR`             Indicates the major version number of the Systems Biology Research Tool. |
| `static int` | `MINOR`             Indicates the minor version number of the Systems Biology Research Tool. |
| `static int` | `REVISION`             Indicates the revision number of the Systems Biology Research Tool. |
| `static java.lang.String` | `USAGE`             Indicates the typical way in which this program is called from the command line. |
| `static java.lang.String` | `VERSION_ARG`             The argument used to display the version number. |


**Constructor Summary**

`Sbrt()`


| **Method Summary** | |
| --- | --- |
| `static java.lang.String` | `getVersion()`             Returns the full version number of the Systems Biology Research Tool. |
| `static void` | `main(java.lang.String[] args)`             Parses the provided command line arguments and executes the application they specify. |

**Methods inherited from class java.lang.Object**

`clone, equals, finalize, getClass, hashCode, notify, notifyAll, toString, wait, wait, wait`

**Field Detail**

### MAJOR

```
public static final int MAJOR
```

:   Indicates the major version number of the Systems Biology Research Tool.

    **See Also:**: Constant Field Values

---


### MINOR

```
public static final int MINOR
```

:   Indicates the minor version number of the Systems Biology Research Tool.

    **See Also:**: Constant Field Values

---


### REVISION

```
public static final int REVISION
```

:   Indicates the revision number of the Systems Biology Research Tool.

    **See Also:**: Constant Field Values

---


### USAGE

```
public static final java.lang.String USAGE
```

:   Indicates the typical way in which this program is called from the
    command line.

    **See Also:**: Constant Field Values

---


### GUI\_ARG

```
public static final java.lang.String GUI_ARG
```

:   The argument used to launch the GUI from the command line.

    **See Also:**: Constant Field Values

---


### VERSION\_ARG

```
public static final java.lang.String VERSION_ARG
```

:   The argument used to display the version number.

    **See Also:**: Constant Field Values


**Constructor Detail**

### Sbrt

```
public Sbrt()
```


**Method Detail**

### getVersion

```
public static java.lang.String getVersion()
```

:   Returns the full version number of the Systems Biology Research Tool.

    :   **Returns:**: the full version number of the Systems Biology Research Tool.

---


### main

```
public static void main(java.lang.String[] args)
```

:   Parses the provided command line arguments and executes the application
    they specify.

    :   **Parameters:**: `args` - the command line arguments.


---


|  |  |  |
| --- | --- | --- |
| **Overview** \| **Package** \| **Class** \| **Use** \| **Tree** \| **Deprecated** \| **Index** \| **Help** | |  |
| **PREV CLASS**   NEXT CLASS | **FRAMES**    **NO FRAMES**     **All Classes** |  |
| SUMMARY: NESTED \| FIELD \| CONSTR \| METHOD | DETAIL: FIELD \| CONSTR \| METHOD |  |


---
